# Optimal Control of Gene Regulatory Networks with Effectiveness of Multiple Drugs: A Boolean Network Approach

**DOI:** 10.1155/2013/246761

**Published:** 2013-08-21

**Authors:** Koichi Kobayashi, Kunihiko Hiraishi

**Affiliations:** School of Information Science, Japan Advanced Institute of Science and Technology, Ishikawa 923-1292, Japan

## Abstract

Developing control theory of gene regulatory networks is one of the significant topics in the field of systems biology, and it is expected to apply the obtained results to gene therapy technologies in the future. In this paper, a control method using a Boolean network (BN) is studied. A BN is widely used as a model of gene regulatory networks, and gene expression is expressed by a binary value (0 or 1). In the control problem, we assume that the concentration level of a part of genes is arbitrarily determined as the control input. However, there are cases that no gene satisfying this assumption exists, and it is important to consider structural control via external stimuli. Furthermore, these controls are realized by multiple drugs, and it is also important to consider multiple effects such as duration of effect and side effects. In this paper, we propose a BN model with two types of the control inputs and an optimal control method with duration of drug effectiveness. First, a BN model and duration of drug effectiveness are discussed. Next, the optimal control problem is formulated and is reduced to an integer linear programming problem. Finally, numerical simulations are shown.

## 1. Introduction

In the field of systems biology, there have been a lot of studies on modeling, analysis, and control of gene regulatory networks. Especially, control of gene regulatory networks corresponds to therapeutic interventions, which are realized by radiation, chemotherapy, and so on. In order to develop gene therapy technologies (see, e.g., [1]) in the future, developing control theory of gene regulatory networks is important. Furthermore, in recent years, the important result on control of gene regulatory networks has been obtained in [2]. That is, feedback control of synthetic biological circuits has been implemented, and the experimental result in which cellular behavior is regulated by control has been obtained. This result suggests that control methods of gene regulatory networks can be realized. Motivated by the above background, we study control methods of gene regulatory networks.

Gene regulatory networks are in general expressed by ordinary/partial differential equations with high nonlinearity and high dimensionality. In order to deal with such a system, it is important to consider a simple model, and various models such as Bayesian networks, Boolean networks (BNs) [3], hybrid systems (piecewise affine models), and Petri nets have been developed so far (see, e.g., [4] for further details). In control problems, BNs and hybrid systems are frequently used [5–8]. In the hybrid systems-based approach, the class of gene regulatory networks are limited to low-dimensional systems, because the computation time to solve the control problem is too long. In a BN, dynamics such as interactions between genes are expressed by the Boolean functions [3]; that is, gene expression is expressed by a binary value (0 or 1). There is a criticism that a Boolean network is too simple as a model of gene regulatory networks (see, e.g., [9]), but this model can be relatively applied to large-scale systems. In addition, since the behavior of gene regulatory networks is stochastic by the effects of noise, it is appropriate that a Boolean function is randomly decided at each time among the candidates of the Boolean functions. From this viewpoint, a probabilistic Boolean network (PBN) has been proposed in [10]. Furthermore, a context-sensitive PBN (CS-PBN) in which the deciding time is randomly selected has been proposed as a general form of PBNs [11, 12].

Furthermore, in the control theory of gene regulatory networks, the control input is given by the concentration level of a part of genes; that is, we assume that the concentration level of a part of genes can be arbitrarily determined. However, in the case where this assumption is not satisfied, it is important to consider structural control via external stimuli [13, 14]. These controls are realized by multiple drugs, and it is also important to consider multiple effects such as duration of effect and side effects [15]. To our knowledge, a unified method considering these properties has not been proposed so far.

Thus, in this paper, we propose a BN model with two types of the control inputs and an optimal control method with duration of drug effectiveness. The first control input is the control input satisfying the assumption that the binary value is arbitrarily determined. The second control input is called a structural control input herein, and the dynamics, that is, the Boolean functions, are selected among the candidates of the dynamics. However, it is difficult to uniquely select one Boolean function. Hence, we suppose that one Boolean function is selected probabilistically, and the probability distribution is switched by using the structural control input. A structural control method has been discussed in [13, 14], but the notion of the structural control input defined in this paper is different from that in those existing methods. Since the proposed BN model has a switch of the probability distribution, it may be regarded as a generalized version of PBNs.

In optimal control of PBNs and CS-PBNs, many results have been obtained so far (see, e.g., [11, 12, 16–21]). In many existing results, state transition diagrams with 2^*n*^ nodes (i.e., 2^*n*^ × 2^*n*^ transition probability matrices) must be computed for a PBN with *n* states. As a result, in order to compute state transition diagrams, several issues such as memory consumption must be considered in implementation, and it is desirable to directly use a given Boolean function. The authors have proposed in [22] a control method in which state transition diagrams are not computed. In many existing results, we consider finding a control input such that the expected value of the cost function is minimized. In [22], we consider finding a control input such that the lower bound of the cost function is minimized under a certain constraint condition. Owing to this difference, state transition diagrams are not computed in the method in [22], and the optimal control problem is reduced to an integer linear programming (ILP) problem. Also in [16], ILP-based methods were proposed for other optimal control problems, and in those methods, solving multiple ILP problems is required.

In this paper, based on our previously proposed method [22], the optimal control problem with duration of drug effectiveness is reduced to an ILP problem. Since a given Boolean function is directly used, duration of drug effectiveness can be easily described as a linear inequality constraint. The proposed method provides us with a basic in control theory of gene regulatory networks. The conference paper [23] is a preliminary version of this paper. In this paper, we provide improved formulations and explanations, a discussion on duration of drug effectiveness, and a numerical simulation using the large-scale BN.

This paper is organized as follows. In Section 2.1, the Boolean networks with two kinds of the control inputs are proposed. In Section 2.2, duration of drug effectiveness is introduced. In Section 2.3, the optimal control problem is formulated. In Section 2.4, its solution method is proposed. In Section 3, two numerical examples are presented. In Section 4, we conclude this paper.

**Notation 1**

Let *ℛ* denote the set of real numbers. Let {0,1}^*n*^ denote the set of *n*-dimensional vectors, which consists of elements 0 and 1. Let *I*_*n*_ and 0_*m*×*n*_ denote the *n* × *n* identity matrix and the *m* × *n* zero matrix, respectively. For simplicity, we sometimes use the symbol 0 instead of 0_*m*×*n*_ and and the symbol *I* instead of *I*_*n*_. For a matrix *M*, ln⁡⁡*M* denotes the matrix such that the (*i*, *j*)th element is given as the natural logarithm of the (*i*, *j*)th element in *M*. For a matrix *M*, *M* ^*T*^ denotes the transpose of *M*.

## 2. Materials and Methods

### 2.1. The Boolean Networks with Control Inputs

A Boolean network (BN) with *n* states is given by

(1) $$\begin{array}{c} x\left(k+1\right)=f_{a}\left(x\left(k\right)\right), \end{array}$$

where *x* ∈ {0,1}^*n*^ is the state (e.g., the concentration of genes) and *k* = 0,1, 2,… is the discrete time. The function *f*_*a*_ : {0,1}^*n*^ → {0,1}^*n*^ is a given Boolean function with logical operators such as AND (∧), OR (∨), and NOT (¬). If the BN (1) is deterministic, then the next state *x*(*k* + 1) is uniquely determined for a given *x*(*k*). See also Example 1 for an example.

Next, the control inputs are added to a BN (1). For the BN (1) with *n* state, consider two types of the control inputs. First, in a similar way to that of the conventional control method, the control input is added to the BN (1) as follows:

(2) $$\begin{array}{c} x\left(k+1\right)=f\left(x\left(k\right),u\left(k\right)\right), \end{array}$$

where *u* ∈ {0,1}^*m*^ is the control input; that is, the value of *u* (e.g., the concentration of genes) can be arbitrarily given, and *f* : {0,1}^*n*^ × {0,1}^*m*^ → {0,1}^*n*^ is a given Boolean function. The *i*th element of the state *x* and the *i*th element of the control input *u* are denoted by *x*_*i*_ and *u*_*i*_, respectively. In the BN (2), *x*(*k* + 1) is uniquely determined for the given *x*(*k*) and *u*(*k*).

Then, consider the structural control input. Suppose that the candidates of *f* are given by *f*_*i*_, *i* = 1,2,…, *l*. It will be difficult to select one Boolean function uniquely. In this paper, we assume that one discrete probability distribution is selected among *m*_*s*_ discrete probability distributions. Probabilistic distributions are derived from experimental results, but details are one of the future works. Then, a method for inferring a probabilistic Boolean network will be useful (see, e.g., [24]). Let *r*_*i*,*j*_ denote the probability that the Boolean function *f*_*j*_ is selected in the *i*th discrete probability distribution. Then,

(3) $$\begin{array}{c} \sum_{j=1}^{l}r_{i,j}=1,\;i=1,2,\ldots ,m_{s}, \end{array}$$

hold. In addition, *m*_*s*_-dimensional binary variables *u* ^*s*^ ∈ {0,1}^*m*_*s*_^ are assigned to *m*_*s*_ discrete probability distributions, and let *u*_*i*_^*s*^ denote the *i*th element of *u* ^*s*^. The structural control input *u* ^*s*^ corresponds to *m*_*s*_ kinds of external stimuli. Then, the equality constraint

(4) $$\begin{array}{c} \sum_{i=1}^{m_{s}}u_{i}^{s}\left(k\right)=1 \end{array}$$

is imposed. Here, we show a simple example.

**Example 1**

**Figure 1:**
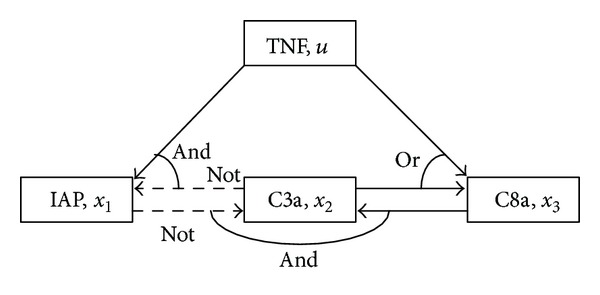
A simplified model of an apoptosis network: activation (solid) and inhibition (broken).

As a simple example, consider the simplified model of an apoptosis network in Figure 1 [25]. Then, the Boolean network model expressing this apoptosis network is given by

(5) $$\begin{array}{c} x_{1}\left(k+1\right)=\neg x_{2}\left(k\right)\wedge u\left(k\right),\\ x_{2}\left(k+1\right)=\neg x_{1}\left(k\right)\wedge x_{3}\left(k\right),\\ x_{3}\left(k+1\right)=x_{2}\left(k\right)\vee u\left(k\right), \end{array}$$

where the concentration level (high or low) of the inhibitor of apoptosis proteins (IAPs) is denoted by *x*_1_, the concentration level of the active caspase 3 (C3a) is denoted by *x*_2_, and the concentration level of the active caspase 8 (C8a) is denoted by *x*_3_. The concentration level of the tumor necrosis factor (TNF, a stimulus) is denoted by *u* and is regarded as the control input. Since the caspase C3a is responsible for cleaving or breaking many other proteins, a high level of the C3a concentration, that is, *x*_2_ = 1, implies cell near-death, otherwise, cell survival. As seen in (5), if the concentration of IAP is high (*x*_1_ = 1) or the concentration of the caspase C8a is low (*x*_3_ = 0), then the concentration of C3a becomes low; that is, *x*_2_ = 0. On the other hand, *x*_1_ and *x*_3_ at the next time depend on the value of *x*_2_ as well as *u*. In this way, some dynamical interactions exist. See [25, 26] for further details.

Suppose that *l* = 2 and *m*_*s*_ = 2. Then, as an example of the candidates of the Boolean functions, we consider the following:

(6) $$\begin{array}{c} f_{1}=\left[\begin{array}{c} \neg x_{2}\left(k\right)\wedge u\left(k\right)\\ \neg x_{1}\left(k\right)\wedge x_{3}\left(k\right)\\ x_{2}\left(k\right)\vee u\left(k\right) \end{array}\right],\;r_{1,1}=0.8,\;r_{2,1}=0.1,\\ f_{2}=\left[\begin{array}{c} x_{1}\left(k\right)\\ x_{2}\left(k\right)\\ x_{3}\left(k\right) \end{array}\right],\;r_{1,2}=0.2,\;r_{2,2}=0.9. \end{array}$$

We suppose that the Boolean function *f*_1_ expresses the situation that the dynamics of an apoptosis network are selected with high probability and that the Boolean function *f*_2_ expresses the situation that the state is not changed with high probability. By using *u*_1_^*s*^ and *u*_2_^*s*^, one of the two discrete probability distributions {*r*_1,1_, *r*_1,2_} and {*r*_2,1_, *r*_2,2_} is selected at each time.

A BN with two types of the control inputs includes the probabilistic behavior, and we assume that the probability distribution can be controlled. From these facts, a BN studied in this paper can be regarded as a generalized form of a probabilistic Boolean network (PBN). To explain the relation between the proposed BN model and a PBN, we show a simple example.

**Example 2**

As a simple example, consider the PBN with three states and one control input. Suppose that the Boolean functions are given as follows:

(7) $$\begin{array}{c} x_{1}\left(k+1\right)=\{\begin{array}{cc} x_{3}\left(k\right)\vee u\left(k\right), & \text{with}\;\text{the}\;\text{probability}\;0.8,\\ \neg x_{3}\left(k\right), & \text{with}\;\text{the}\;\text{probability}\;0.2, \end{array}\\ x_{2}\left(k+1\right)=x_{1}\left(k\right)\wedge \neg x_{3}\left(k\right),\text{with}\;\text{the}\;\text{probability}\;1.0,\\ x_{3}\left(k+1\right)=\{\begin{array}{cc} x_{1}\left(k\right)\wedge \neg x_{2}\left(k\right), & \text{with}\;\text{the}\;\text{probability}\;0.7,\\ x_{2}\left(k\right)\vee u\left(k\right), & \text{with}\;\text{the}\;\text{probability}\;0.3. \end{array} \end{array}$$

This PBN corresponds to the cases of *l* = 4 and *m*_*s*_ = 1. The candidates of the Boolean functions *f*_*i*_, *i* = 1,2, 3,4, and the probabilities *r*_1,*j*_, *j* = 1,2, 3,4, are obtained as follows:

(8) $$\begin{array}{c} f_{1}=\left[\begin{array}{c} x_{3}\left(k\right)\vee u\left(k\right)\\ x_{1}\left(k\right)\wedge \neg x_{3}\left(k\right)\\ x_{1}\left(k\right)\wedge \neg x_{2}\left(k\right) \end{array}\right],\;r_{1,1}=0.56,\\ f_{2}=\left[\begin{array}{c} x_{3}\left(k\right)\vee u\left(k\right)\\ x_{1}\left(k\right)\wedge \neg x_{3}\left(k\right)\\ x_{2}\left(k\right)\vee u\left(k\right) \end{array}\right],\;r_{1,2}=0.24,\\ f_{3}=\left[\begin{array}{c} \neg x_{3}\left(k\right)\\ x_{1}\left(k\right)\wedge \neg x_{3}\left(k\right)\\ x_{1}\left(k\right)\wedge \neg x_{2}\left(k\right) \end{array}\right],\;r_{1,3}=0.14,\\ f_{4}=\left[\begin{array}{c} \neg x_{3}\left(k\right)\\ x_{1}\left(k\right)\wedge \neg x_{3}\left(k\right)\\ x_{2}\left(k\right)\vee u\left(k\right) \end{array}\right],\;r_{1,4}=0.06. \end{array}$$

Next, consider the state orbit of this PBN. In PBNs, one Boolean function is probabilistically selected at each time. Then, for $x(0)={\left[\begin{array}{ccc} 0 & 0 & 0 \end{array}\right]}^{T}$ and *u*(0) = 0, we obtain the following:

(9) $$\begin{array}{c} \text{Prob}\left(x\left(1\right)={\left[\begin{array}{ccc} 0 & 0 & 0 \end{array}\right]}^{T}\;|\;x\left(0\right)={\left[\begin{array}{ccc} 0 & 0 & 0 \end{array}\right]}^{T}\right)=0.8,\\ \text{Prob}\left(x\left(1\right)={\left[\begin{array}{ccc} 1 & 0 & 0 \end{array}\right]}^{T}\;|\;x\left(0\right)={\left[\begin{array}{ccc} 0 & 0 & 0 \end{array}\right]}^{T}\right)=0.2. \end{array}$$

**Figure 2:**
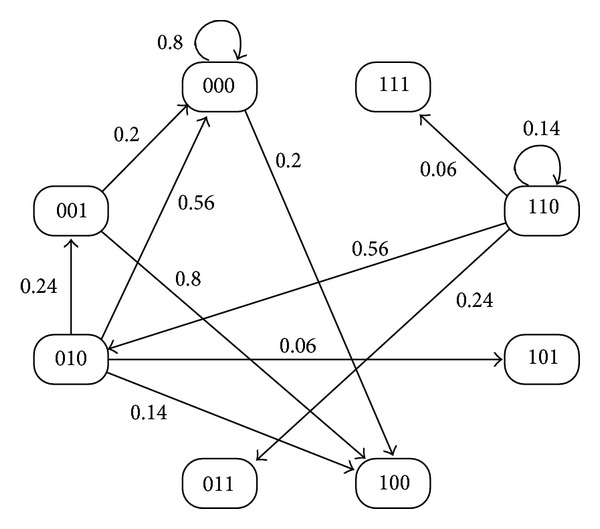
The state transition diagram with *u*(*k*) = 0.

In this example, the cardinality of the finite state set {0,1}^3^ is given by 2^3^ = 8, and we obtain the state transition diagram of Figure 2 by computing the transition from each value of the state. In Figure 2, the number assigned to each node denotes *x*_1_, *x*_2_, and *x*_3_ (each element of the state), and the number assigned to each arc denotes the transition probability from some state to another state. For simplicity of illustration, the state transitions from $x(k)={\left[\begin{array}{ccc} 0 & 0 & 0 \end{array}\right]}^{T},{\left[\begin{array}{ccc} 0 & 0 & 1 \end{array}\right]}^{T},{\left[\begin{array}{ccc} 0 & 1 & 0 \end{array}\right]}^{T},{\left[\begin{array}{ccc} 1 & 1 & 0 \end{array}\right]}^{T}$ are illustrated in Figure 2. In the existing solution methods for optimal control of PBNs, the optimal control input is computed using dynamic programming with state transition diagrams.

As shown in this example, computing state transition diagrams with 2^*n*^ nodes (*n* is the number of the state) is required in the existing solution methods for optimal control of PBNs with *n* states, and this computation is hard for large-scale systems (see also Section 3.2). Thus, it is important to consider a new solution method. In this paper, for BNs with two types of the control inputs, a solution method using integer programming is proposed based on our previously proposed work in [22]. In the proposed method, computation of state transition diagrams such as that in Figure 2 is not needed.

**Remark 3**

By adding the candidates of the Boolean functions, BNs with two types of the control inputs can be transformed into BNs with only the structural control input. That is, the control input *u* can be eliminated from (2) by fixing the value of *u* in (2). Then, the number of the candidates of Boolean functions is 2^*m*^ *l*, and 2^*m*^ combinations for *u* must be computed in advance. To avoid this computation, we consider two types of the control inputs.

### 2.2. Duration of Drug Effectiveness

The control input *u* and the structural control input *u* ^*s*^ are realized by using multiple drugs. Then, we must consider the multiple effects such as duration of effect and the side effects. In this paper, we focus on the duration of drug effectiveness. In, for example, chemotherapy, therapeutic intervention is generally applied to the target cell in a cyclic manner [15]. Each therapeutic window is started by delivering the drug. The drug delivered is effective on the target cell for some period of time. This is followed by a recovery phase. However, when the drug is not delivered, the drug may be delivered in the timing that is faster than the next time in a cyclic [15]. Therefore, it is necessary to model several situations on duration of drug effectiveness. To model the duration of effect, three parameters *L*_*u*_*i*__, *W*_*u*_*i*__^1^, and *W*_*u*_*i*__^0^ are defined for each input *u*_*i*_ (or *u*_*i*_^*s*^). The parameters *L*_*u*_*i*__ and *W*_*u*_*i*__^1^ have been already defined in [15].

The parameter *L*_*u*_*i*__ is the length of the drug effectiveness period. That is, if *u*_*i*_(*k*) = 1, then *u*_*i*_(*k* + 1) = *u*_*i*_(*k* + 2) = ⋯ = *u*_*i*_(*k* + *L*_*u*_*i*__) = 1 holds. Next, *W*_*u*_*i*__^1^(>*L*_*u*_*i*__) is explained. If *u*_*i*_(*k*) = 1, then *u*_*i*_(*k* + 1), *u*_*i*_(*k* + 2),…, *u*_*i*_(*k* + *W*_*u*_*i*__^1^ − 1) is uniquely determined depending on *L*_*u*_*i*__, and *u*_*i*_(*k* + *W*_*u*_*i*__^1^) is a decision variable. Then, *W*_*u*_*i*__^1^ − *L*_*u*_*i*__ − 1 corresponds to the length of a recovery phase. Finally, *W*_*u*_*i*__^0^ is explained. If *u*_*i*_(*k*) = 0, then *u*_*i*_(*k* + 1) = *u*_*i*_(*k* + 2) = ⋯ = *u*_*i*_(*k* + *W*_*u*_*i*__^0^ − 1) = 0 holds, and *u*_*i*_(*k* + *W*_*u*_*i*__^0^) is a decision variable. By using *L*_*u*_*i*__, *W*_*u*_*i*__^1^, *W*_*u*_*i*__^0^, we can consider several situations, and we show two typical examples.

**Example 4**

First, suppose that, for the control input *u* ∈ {0,1}^1^, *L*_*u*_, *W*_*u*_^1^, and *W*_*u*_^0^ are given as *L*_*u*_ = 1, *W*_*u*_^1^ = 3, and *W*_*u*_^0^ = 2, respectively. Consider the case of *u*(*k*) = 1. Then, *u*(*k* + 1) and *u*(*k* + 2) are uniquely determined as *u*(*k* + 1) = 1 and *u*(*k* + 2) = 0, respectively, and *u*(*k* + 3) is a decision variable. Then, *u*(*k* + 2) = 0 is the recovery phase, and *W*_*u*_^1^ − *L*_*u*_ − 1 = 1 is its length. In the case of *u*(*k*) = 0, the relation *u*(*k* + 1) = 0 holds, and *u*(*k* + 2) is a decision variable.

Another example is shown. Suppose that, for the control input *u* ∈ {0,1}^1^, *L*_*u*_, *W*_*u*_^1^, and *W*_*u*_^0^ are given as *L*_*u*_ = 0, *W*_*u*_^1^ = 3, and *W*_*u*_^0^ = 3, respectively. In both, the case of *u*(*k*) = 1 and the case of *u*(*k*) = 0, *u*(*k* + 1) = *u*(*k* + 2) = 0 holds, and *u*(*k* + 3) is a decision variable. In this case, *u*(*k* + 1) = *u*(*k* + 2) = 0 is the recovery phase, and *W*_*u*_^1^ − *L*_*u*_ − 1 = 2 is its length.

By using the three parameters *L*_*u*_*i*__, *W*_*u*_*i*__^1^, and *W*_*u*_*i*__^0^, several situations on the duration of effect can be modeled (see also [15]). In addition, since these parameters can be given for each *u*_*i*_ (or *u*_*i*_^*s*^), effectiveness of multiple drugs can be evaluated. Thus, in this paper, we consider not only two types of the control inputs but also duration of drug effectiveness.

### 2.3. Optimal Control Problem

First, the following two notations are defined. Let *π*_*i*_(*k*) denote the probability that some Boolean function *f*_*i*_ is selected at time *k*. In addition, the probability that some time sequence of theBoolean functions *f*_*i*(*k*_1_)_, *f*_*i*(*k*_1_+1)_,…, *f*_*i*(*k*_2_)_ is selected at time interval [*k*_1_, *k*_2_] is denoted by

(10) $$\begin{array}{c} \pi \left(k_{1},k_{2}\right):=\prod_{k=k_{1}}^{k_{2}}\pi_{i(k)}\left(k\right). \end{array}$$

For simplicity of notation, *i*(*k*_1_), *i*(*k*_1_ + 1),…, *i*(*k*_2_) are omitted in *π*(*k*_1_, *k*_2_).

Next, for the Boolean networks with *n* states and two types of the control inputs, consider the following optimal control problem.

**Problem 1**

Suppose that, for the Boolean network with *n* states and two types of the control inputs, the initial state *x*(0) = *x*_0_, *ρ* satisfying 0 ≤ *ρ* ≤ 1, the control time *N*, the parameters on duration of drug effectiveness *L*_*u*_*i*_(*u*_*i*_^*s*^)_, *W*_*u*_*i*_(*u*_*i*_^*s*^)_^1^, and *W*_*u*_*i*_(*u*_*i*_^*s*^)_^0^ are given. Then, for all combinations of the Boolean functions satisfying the constraint

(11) $$\begin{array}{c} \pi \left(0,N-1\right)\geq \rho , \end{array}$$

find two control input sequences *u*(0), *u*(1),…, *u*(*N* − 1) and *u* ^*s*^(0), *u* ^*s*^(1),…, *u* ^*s*^(*N* − 1) minimizing the lower bound of the cost function

(12) $$\begin{array}{c} J=\sum_{k=0}^{N-1}\left\{Qx\left(k\right)+Ru\left(k\right)+R_{s}u^{s}\left(k\right)\right\}+Q_{f}x\left(N\right) \end{array}$$

subject to the constraint on duration of drug effectiveness, where *Q*, *Q*_*f*_ ∈ *ℛ* ^1×*n*^, *R* ∈ *ℛ* ^1×*m*^, and *R* ∈ *ℛ* ^1×*m*_*s*_^ are weighting vectors whose elements are nonnegative real numbers.

For simplicity of discussion, a linear function with respect to *x*, *u*, and *u* ^*s*^ is considered as a cost function. We consider that a linear cost function is appropriate from the following two reasons.

- For a binary variable *δ* ∈ {0,1}, the relation *δ* ^2^ = *δ* holds. That is, in the cost function, the quadratic term such as *x*_*i*_^2^(*k*) is not necessary.
- In control of gene regulatory networks, the expression of a certain gene is frequently focused (see, e.g., [18]). For example, in the gene regulatory network related to melanoma, it important to inhibit the concentration level of the gene WNT5A [27]. In this case, it is enough to consider the cost function (12).

Furthermore, in many existing methods on optimal control of PBNs, the expected value of a nonnegative function is frequently used as a cost function (see, e.g., [11, 12, 17–21]). However, the expected value of the state must be computed from all combinations of the Boolean functions, and this computation is hard for large-scale systems. To avoid this computation, in this paper, we evaluate the control performance by using the lower bound. If the constraint (11) is not included in Problem 1, then the behaviors are regarded as uncertain (nondeterministic) behaviors, and the best performance is derived in Problem 1. Since the combinations of the Boolean functions selected with low probability are included, performance evaluation is not appropriate. In order to exclude such combinations, we impose the constraint (11). Similar problem formulations have been considered in optimal control of stochastic hybrid systems (see, e.g., [28–30]). Thus, since the performance index in this paper is different from that in existing methods, it is difficult to directly compare the performance of the proposed method with those of existing methods. On the other hand, in [22], we discussed this topic from the qualitative viewpoint. In [22], the upper bound is also computed by using the control input such that the lower bound is minimized. If the lower bound and the upper bound are not improved by control, then the expected value will not be improved. Then, it is important to suitably set *ρ* in the constraint (11). See [22] for further details.

We show an example for setting weighting vectors from the biological viewpoint.

**Example 5**

Consider the Boolean network expressing an apoptosis network in Example 1 again. For this system, we consider finding a control strategy such that a stimulus *u* is not applied as much as possible, and cell survival is achieved; *u* = 0 implies that a stimulus is not applied to the system, and *x*_1_ = 1 and *x*_2_ = 0 express cell survival [25]. Then, as one of the appropriate cost functions, we can consider the following cost function:

(13) J=∑i=0N−1{10|x1(i)−1|+10|x2(i)−0|+u(i)}+100|x1(N)−1|+100|x2(N)−0|.

By the coordinate transformation of *x*_1_ into 1 − *x*_1_, this cost function can be rewritten as the form of (12).

### 2.4. Solution Method

We propose a solution method for Problem 1. First, two lemmas are introduced as preparations. Next, Problem 1 is reduced to an integer linear programming (ILP) problem.

As preparations, two lemmas are introduced. To reduce Problem 1 to an ILP problem, it is necessary to transform a Boolean function into a polynomial on the real number field. First, the following lemma [31] is used.

**Lemma 6**

Consider the two binary variables *δ*_1_, and *δ*_2_. Then, the following relations hold:

- ¬*δ*_1_ is equivalent to 1 − *δ*_1_,
- *δ*_1_∨*δ*_2_ is equivalent to *δ*_1_ + *δ*_2_ − *δ*_1_ *δ*_2_,
- *δ*_1_∧*δ*_2_ is equivalent to *δ*_1_ *δ*_2_.

For example, *δ*_1_∨¬*δ*_2_ is equivalently transformed into *δ*_1_ + (1 − *δ*_2_) − *δ*_1_(1 − *δ*_2_) = 1 − *δ*_2_ + *δ*_1_ *δ*_2_. Furthermore, the product of binary variables such as *δ*_1_ *δ*_2_ can be linearized by using the following lemma [32].

**Lemma 7**

Suppose that the binary variables *δ*_*j*_ ∈ {0,1} and *j* ∈ *𝒥* are given, where *𝒥* is some index set. Then, *z* = ∏_*j*∈*𝒥*_ *δ*_*j*_ is equivalent to the following linear inequalities:

(14) $$\begin{array}{c} \sum_{j\in 𝒥}\delta_{j}-z\leq \left|𝒥\right|-1,\;\;-\sum_{j\in 𝒥}\delta_{j}+\left|𝒥\right|z\leq 0, \end{array}$$

where |*𝒥*| is the cardinality of *𝒥*.

From Lemmas 6 and 7, we see that any Boolean function can be equivalently transformed into a pair of some linear function and some linear inequality. See [31, 32] for further details. For example, *δ*_1_∨¬*δ*_2_ is equivalent to a pair of 1 − *δ*_2_ + *z* and *z* = *δ*_1_ *δ*_2_. By using Lemma 7, *z* = *δ*_1_ *δ*_2_ can be expressed as a set of linear inequalities.

Now, we consider reducing Problem 1 to an ILP problem.

By using Lemma 6, the candidates of the Boolean functions *f*_*i*_(*x*(*k*), *u*(*k*)), *i* = 1,2,…, *l*, are transformed into a polynomial on the real number field. Let ${\hat{f}}_{i}(x(k),u(k))$ denote the polynomial obtained. Then, consider the following system using ${\hat{f}}_{i}(x(k),u(k))$:

(15) $$\begin{array}{c} x\left(k+1\right)=\sum_{i=1}^{l}\left\{\delta_{i}\left(k\right){\hat{f}}_{i}\left(x\left(k\right),u\left(k\right)\right)\right\}, \end{array}$$

where *δ*_1_(*k*), *δ*_2_(*k*),…, *δ*_*l*_(*k*) are binary variables satisfying

(16) $$\begin{array}{c} \sum_{i=1}^{l}\delta_{i}\left(k\right)=1. \end{array}$$

The binary vector $\delta (k):={\left[\begin{array}{cccc} \delta_{1}\left(k\right) & \delta_{2}\left(k\right) & \cdots & \delta_{l}\left(k\right) \end{array}\right]}^{T}$ is used to select the polynomial ${\hat{f}}_{i}$ and to express (11) as a linear form. Here, we define the following vector:

(17) $$\begin{array}{c} S_{i}:=\left[\begin{array}{cccc} r_{i,1} & r_{i,2} & \cdots & r_{i,l} \end{array}\right]. \end{array}$$

Then, by using the natural logarithm, *π*(0, *N* − 1) in (11) is expressed as

(18) $$\begin{array}{c} \ln\pi \left(0,N-1\right)=\sum_{k=0}^{N-1}\left(\sum_{i=1}^{m_{s}}\ln S_{i}u_{i}^{s}\left(k\right)\right)\delta \left(k\right). \end{array}$$

In this expression, one probability distribution is selected by using *u*_*i*_^*s*^(*k*), and the probability that a certain Boolean function is selected is determined by *δ*(*k*). Then, Problem 1 is equivalent to the following problem.


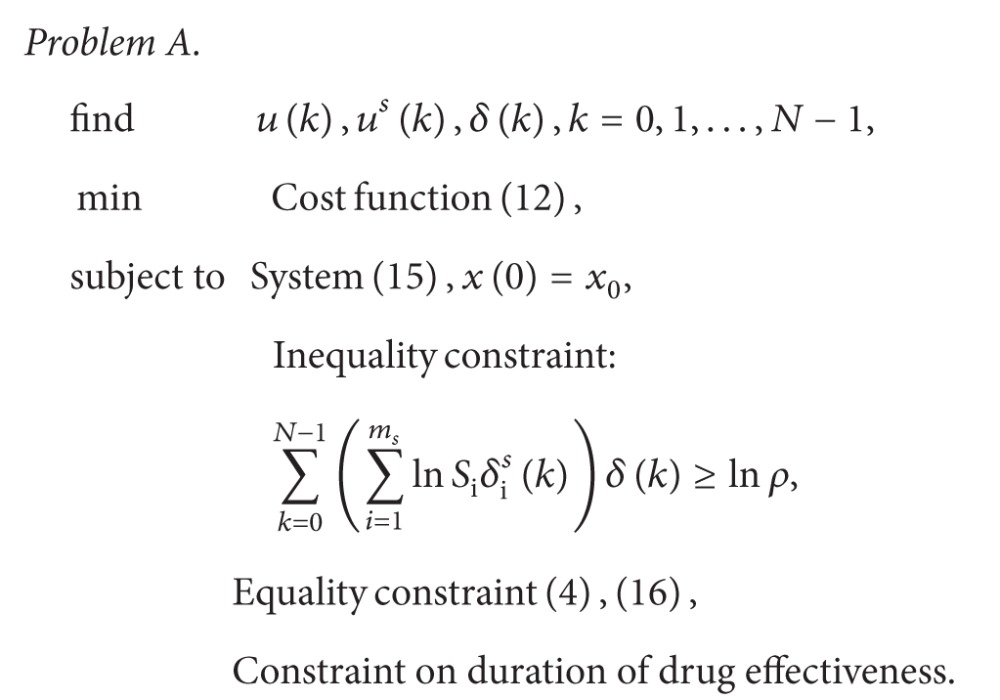
(19)

By using Lemma 7, the system (15) and (∑_*i*=1_^*m*_*s*_^ln⁡⁡*S*_*i*_ *δ*_*i*_^*s*^(*k*))*δ*(*k*) can be equivalently expressed in the following linear form:

(20) $$\begin{array}{c} x\left(k+1\right)=Ax\left(k\right)+B_{u}u\left(k\right)+B_{s}u^{s}\left(k\right)+B_{b}z_{b}\left(k\right), \end{array}$$

(21) $$\begin{array}{c} \left(\sum_{i=1}^{m_{s}}\ln S_{i}\delta_{i}^{s}\left(k\right)\right)\delta \left(k\right)=\sum_{i=1}^{m_{s}}\ln S_{i}z_{i}^{s}\left(k\right), \end{array}$$

(22) $$\begin{array}{c} Ex\left(k\right)+F_{u}u\left(k\right)+F_{s}u^{s}\left(k\right)+F_{z}z\left(k\right)\leq G, \end{array}$$

where *z*_*i*_^*s*^(*k*) : = *δ*_*i*_^*s*^(*k*)*δ*(*k*), and (22) is the linear inequality obtained by applying Lemma 7 to (15). The vector *z*_*b*_(*k*)∈{0,1}^*b*^ is an auxiliary binary variable obtained by using Lemma 7, and the dimension of *z*_*b*_(*k*), that is, *b*, is determined depending on the form of the given Boolean functions. In addition, *z*(*k*) is defined as

(23) z(k)≔[(zb(k))T(z1s(k))T(z2s(k))T⋯(zmss(k))T]T∈{0,1}b+msl.

Here in after, for simplicity of notation, *B*_*b*_ *z*_*b*_(*k*) is rewritten as *B*_*z*_ *z*(*k*), $B_{z}:=\left[\begin{array}{cc} B_{b} & 0 \end{array}\right]$, and ∑_*i*=1_^*m*_*s*_^ln⁡⁡*S*_*i*_ *z*_*i*_^*s*^(*k*) is rewritten as *Cz*(*k*), $C:=\left[\begin{array}{ccccc} 0 & \ln S_{1} & \ln S_{2} & \cdots & \ln S_{m_{s}} \end{array}\right]$.

Now, we consider transforming Problem A by using (20), (21), and (22). By using

(24) $$\begin{array}{c} x\left(k\right)=A^{k}x_{0}+\sum_{i=1}^{k}A^{i-1}\left(B_{u}u\left(k-i\right)+B_{s}u^{s}\left(k-i\right)+B_{z}z\left(k\right)\right) \end{array}$$

obtained from the state equation in (20), we can obtain

(25) $$\begin{array}{c} \bar{x}=\bar{A}x_{0}+{\bar{B}}_{u}\bar{u}+{\bar{B}}_{s}{\bar{u}}_{s}+{\bar{B}}_{z}\bar{z}, \end{array}$$

where

(26) $$\begin{array}{c} \bar{x}:={\left[\begin{array}{cccc} {\left(x\left(0\right)\right)}^{T} & {\left(x\left(1\right)\right)}^{T} & \cdots & {\left(x\left(N\right)\right)}^{T} \end{array}\right]}^{T},\\ \bar{u}:=\;{\left[\begin{array}{cccc} {\left(u\left(0\right)\right)}^{T} & {\left(u\left(1\right)\right)}^{T} & \cdots & {\left(u\left(N-1\right)\right)}^{T} \end{array}\right]}^{T},\\ {\bar{u}}_{s}:={\left[\begin{array}{cccc} {\left(u^{s}\left(0\right)\right)}^{T} & {\left(u^{s}\left(1\right)\right)}^{T} & \cdots & {\left(u^{s}\left(N-1\right)\right)}^{T} \end{array}\right]}^{T},\\ \bar{z}:={\left[\begin{array}{cccc} {\left(z\left(0\right)\right)}^{T} & {\left(z\left(1\right)\right)}^{T} & \cdots & {\left(z\left(N-1\right)\right)}^{T} \end{array}\right]}^{T},\\ \bar{A}=\left[\begin{array}{c} I\\ A\\ A^{2}\\ \vdots \\ A^{N} \end{array}\right],\;\;\bar{B}=\left[\begin{array}{cccc} 0 & 0 & \cdots & 0\\ I & 0 & \cdots & 0\\ A & \ddots & \ddots & \vdots \\ \vdots & \ddots & \ddots & 0\\ A^{N-1} & \cdots & A & I \end{array}\right],\\ {\bar{B}}_{u}=\bar{B}\left[\begin{array}{ccc} B_{u} & & 0\\ & \ddots & \\ 0 & & B_{u} \end{array}\right],\;\;{\bar{B}}_{s}=\bar{B}\left[\begin{array}{ccc} B_{s} & & 0\\ & \ddots & \\ 0 & & B_{s} \end{array}\right],\\ {\bar{B}}_{z}=\bar{B}\left[\begin{array}{ccc} B_{z} & & 0\\ & \ddots & \\ 0 & & B_{z} \end{array}\right]. \end{array}$$

Next, the inequality constraint ∑_*k*=0_^*N*−1^(∑_*i*=1_^*m*_*s*_^ln⁡⁡*S*_*i*_ *δ*_*i*_^*s*^(*k*))*δ*(*k*) ≥ ln⁡⁡*ρ* in Problem A is equivalent to

(27) $$\begin{array}{c} -\bar{C}\bar{z}\leq -\ln\rho , \end{array}$$

where $\bar{C}=\left[\begin{array}{cccc} C & C & \cdots & C \end{array}\right]$. Furthermore, from (22), we can obtain

(28) $$\begin{array}{c} \bar{E}\bar{x}+{\bar{F}}_{u}\bar{u}+{\bar{F}}_{s}{\bar{u}}_{s}+{\bar{F}}_{z}\bar{z}\leq \bar{G}, \end{array}$$

where

(29) $$\begin{array}{c} \bar{E}=\left[\begin{array}{cccc} E & & 0 & 0\\ & \ddots & & \vdots \\ 0 & & E & 0 \end{array}\right],\\ {\bar{F}}_{u}=\left[\begin{array}{ccc} F_{u} & & 0\\ & \ddots & \\ 0 & & F_{u} \end{array}\right],\;\;{\bar{F}}_{s}=\left[\begin{array}{ccc} F_{s} & & 0\\ & \ddots & \\ 0 & & F_{s} \end{array}\right],\\ {\bar{F}}_{z}=\left[\begin{array}{ccc} F_{z} & & 0\\ & \ddots & \\ 0 & & F_{z} \end{array}\right],\;\;\bar{G}=\left[\begin{array}{c} G\\ G\\ \vdots \\ G \end{array}\right]. \end{array}$$

Next, consider the constraint on duration of drug effectiveness. This constraint can be expressed as a Boolean function. Then, by using Lemmas 6 and 7, it can be transformed into the following form:

(30) $$\begin{array}{c} \bar{u}=V^{1}\bar{v}+V^{2},\;\;W^{1}\bar{v}\leq W^{2}, \end{array}$$

(31) $$\begin{array}{c} {\bar{u}}_{s}=V_{s}^{1}{\bar{v}}_{s}+V_{s}^{2},\;\;W_{s}^{1}{\bar{v}}_{s}\leq W_{s}^{2}, \end{array}$$

where $\bar{v}$ and ${\bar{v}}_{s}$ are binary decision variables with certain dimensions. Deriving a general form of coefficient matrices will be difficult, but for the given *L*_*u*_*i*__, *W*_*u*_*i*__^1^, and *W*_*u*_*i*__^0^, deriving coefficient matrices is easy.

We show two examples.

**Example 8**

Consider Example 4 again. First, consider the case of *L*_*u*_ = 1, *W*_*u*_^1^ = 3, and *W*_*u*_^0^ = 2. Then, noting explanations in Example 4, we can obtain

(32) u(0)=v0,u(1)=v0,u(2)=(1−v0)v2,u(3)=(1−v0)v2+v0v3,u(4)=v0v3+(1−v2)v4,u(5)=(1−v2)v4+v2v5,  ⋮

and in this case, these are equivalent to

(33) u(0)=v0,u(1)=v0,u(2)=v2, v2≤1−v0,u(3)=v2+v3, v2≤1−v0, v3≤v0,u(4)=v3+v4, v3≤v0, v4≤1−v2, 0≤v3+v4≤1,u(5)=v4+v5, v4≤1−v2, v2≤v5, 0≤v4+v5≤1,   ⋮

We explain *u*(2) = *v*_2_, and *v*_2_ ≤ 1 − *v*_0_ as an example. If *v*_0_ = 1, then *v*_2_ ≤ 0 holds. Since *v*_2_ is binary, we can obtain *v*_2_ = 0; that is, *u*(2) = 0. If *v*_0_ = 0, then *v*_2_ ≤ 1 holds, and we can obtain *u*(2) = *v*_2_. That is, *u*(2) can take on either 0 or 1. From the previous discussion, we see that a pair of *u*(2) = *v*_2_ and *v*_2_ ≤ 1 − *v*_0_ is equivalent to *u*(2) = (1 − *v*_0_)*v*_2_. Thus, we can obtain the forms of (30) and (31). In the case of *N* = 5 (*N* is the control time in Problem 1), (30) can be obtained as

(34) $$\begin{array}{c} \underset{\bar{u}}{\underset{︸}{\left[\begin{array}{c} u\left(0\right)\\ u\left(1\right)\\ u\left(2\right)\\ u\left(3\right)\\ u\left(4\right) \end{array}\right]}}=\underset{V^{1}}{\underset{︸}{\left[\begin{array}{cccc} 1 & 0 & 0 & 0\\ 1 & 0 & 0 & 0\\ 0 & 1 & 0 & 0\\ 0 & 1 & 1 & 0\\ 0 & 0 & 1 & 1 \end{array}\right]}}\underset{\bar{v}}{\underset{︸}{\left[\begin{array}{c} v_{0}\\ v_{2}\\ v_{3}\\ v_{4} \end{array}\right]}},\\ \underset{W^{1}}{\underset{︸}{\left[\begin{array}{cccc} 1 & 1 & 0 & 0\\ -1 & 0 & 1 & 0\\ 0 & 1 & 0 & 1\\ 0 & 0 & -1 & -1\\ 0 & 0 & 1 & 1 \end{array}\right]}}\underset{\bar{v}}{\underset{︸}{\left[\begin{array}{c} v_{0}\\ v_{2}\\ v_{3}\\ v_{4} \end{array}\right]}}\leq \underset{W^{2}}{\underset{︸}{\left[\begin{array}{c} 1\\ 0\\ 1\\ 0\\ 1 \end{array}\right]}}, \end{array}$$

where *V* ^2^ = 0. We remark that, in a general case, the product such as *z* = *v*_0_ *v*_2_ must be transformed into linear inequalities by using Lemma 7.

Next, consider the case of *L*_*u*_ = 0, *W*_*u*_^1^ = 3, and *W*_*u*_^0^ = 3. Then, we can obtain the following:

(35) u(0)=v0,u(1)=0,u(2)=0,u(3)=v3,u(4)=0,u(5)=0,  ⋮

and this case is one of the simplest cases. In the case of *N* = 5 (*N* is the control time in Problem 1), (30) can be obtained as

(36) $$\begin{array}{c} \underset{\bar{u}}{\underset{︸}{\left[\begin{array}{c} u\left(0\right)\\ u\left(1\right)\\ u\left(2\right)\\ u\left(3\right)\\ u\left(4\right) \end{array}\right]}}=\underset{V^{1}}{\underset{︸}{\left[\begin{array}{cc} 1 & 0\\ 0 & 0\\ 0 & 0\\ 0 & 1\\ 0 & 0 \end{array}\right]}}\underset{\bar{v}}{\underset{︸}{\left[\begin{array}{c} v_{0}\\ v_{3} \end{array}\right]}}, \end{array}$$

where *V* ^2^ = 0, *W* ^1^ = 0, and *W* ^2^ = 0.

Finally, the cost function (12) is rewritten as

(37) $$\begin{array}{c} J=\bar{Q}\bar{x}+\bar{R}\bar{u}+{\bar{R}}_{s}{\bar{u}}_{s}, \end{array}$$

where $\bar{Q}=\left[\begin{array}{cccc} Q & \cdots & Q & Q_{f} \end{array}\right]$, $\bar{R}=\left[\begin{array}{ccc} R & \cdots & R \end{array}\right]$, and ${\bar{R}}_{s}=\left[\begin{array}{ccc} R_{s} & \cdots & R_{s} \end{array}\right]$. By substituting (25), (30), and (31) into (28) and (37), Problem A is equivalent to the following ILP problem.


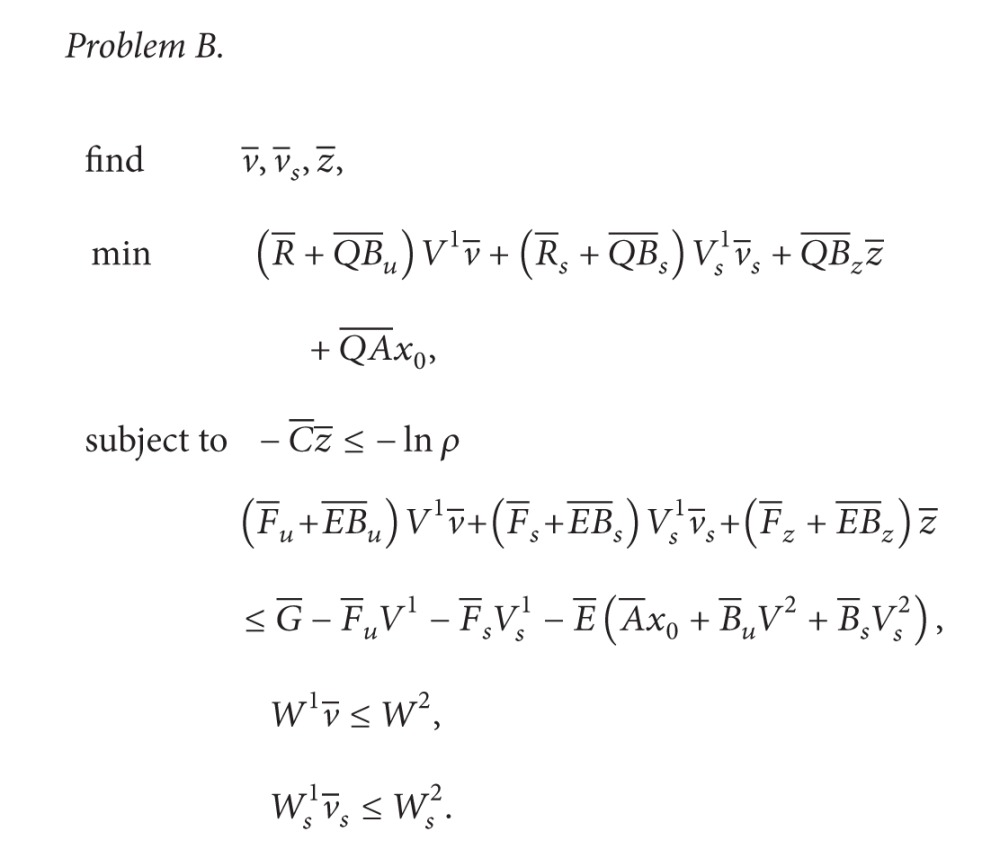
(38)

Problem B can be solved by a suitable solver such as IBM ILOG CPLEX [33].

## 3. Results and Discussion

In this section, we show numerical simulations. First, we consider the WNT5A network [14]. Next, in order to evaluate the proposed method from the viewpoint of the computation time, we consider an artificial example.

### 3.1. WNT5A Network

The gene regulatory network with the gene WNT5A is related to melanoma, and it has been extensively studied (see, e.g., [27]). The BN model *x*(*k* + 1) = *f*_*a*_(*x*(*k*)) of the WNT5A network is given by the following:

(39) x1(k+1)=¬x6(k),x2(k+1)=(¬x2(k)∧x4(k)∧x6(k)) ∨{¬x2(k)∧(x4(k)∨x6(k))},x3(k+1)=¬x7(k),x4(k+1)=x4(k),x5(k+1)=x2(k)∨¬x7(k),x6(k+1)=x3(k)∨x4(k),x7(k+1)=¬x2(k)∨x7(k),

where the concentration level (high or low) of the gene WNT5A is denoted by *x*_1_, the concentration level of the gene pirin by *x*_2_, the concentration level of the gene S100P is denoted by *x*_3_, the concentration level of the gene RET1 is denoted by *x*_4_, the concentration level of the gene MART1 is denoted by *x*_5_, the concentration level of the gene HADHB is denoted by *x*_6_, and the concentration level of the gene STC2 is denoted by *x*_7_. See [14] for further details. In a WNT5A network, it is important to inhibit the concentration level of the gene WNT5A [27].

The optimal control problem is formulated. For simplicity, we consider only the structural control input. Then, suppose that the number of the structural control inputs is two. If *u*_1_^*s*^(*k*) = 1, then the system is given as:

(40) $$\begin{array}{c} x\left(k+1\right)=\{\begin{array}{cc} f_{a}\left(x\left(k\right)\right) & \text{with}\;\text{the}\;\text{probability}\;0.8,\\ x\left(k\right) & \text{with}\;\text{the}\;\text{probability}\;0.2. \end{array} \end{array}$$

If *u*_2_^*s*^(*k*) = 1, then the system is given as:

(41) $$\begin{array}{c} x\left(k+1\right)=\{\begin{array}{cc} f_{a}\left(x\left(k\right)\right) & \text{with}\;\text{the}\;\text{probability}\;0.1,\\ x\left(k\right) & \text{with}\;\text{the}\;\text{probability}\;0.9. \end{array} \end{array}$$

The case of *u*_1_^*s*^(*k*) = 1 corresponds to the situation such that the dynamics of the WNT5A network, that is, *x*(*k* + 1) = *f*_*a*_(*x*(*k*)), are selected with high probability. The case of *u*_2_^*s*^(*k*) = 1 corresponds to the situation such that the state is not changed; that is, *x*(*k* + 1) = *x*(*k*) is selected with high probability. From the previous setting, *r*_1,1_ = 0.8, *r*_1,2_ = 0.2, *r*_2,1_ = 0.1, and *r*_2,2_ = 0.9. For this WNT5A network with structural control inputs, consider solving Problem 1. *Q*, *Q*_*f*_, and *R*_*s*_ in Problem 1 are given as $Q=\left[\begin{array}{ccccccc} 1 & 0 & 0 & 0 & 0 & 0 & 0 \end{array}\right]$, $Q_{f}=\left[\begin{array}{ccccccc} 10 & 0 & 0 & 0 & 0 & 0 & 0 \end{array}\right]$, and $R_{s}=\left[\begin{array}{cc} 0 & 0 \end{array}\right]$, respectively. The initial state is given as $x_{0}={\left[\begin{array}{ccccccc} 1 & 0 & 1 & 0 & 1 & 0 & 0 \end{array}\right]}^{T}$. In addition, the control time *N* in Problem 1 is given by *N* = 5. Finally, the constraint on duration of drug effectiveness is imposed for only *u*_2_^*s*^(*k*). The parameters *L*_*u*_2_^*s*^_, *W*_*u*_2_^*s*^_^1^, and *W*_*u*_2_^*s*^_^0^ are given as *L*_*u*_2_^*s*^_ = 1, *W*_*u*_2_^*s*^_^1^ = 3, and *W*_*u*_2_^*s*^_^0^ = 2, respectively. Thus, we can obtain the ILP problem (Problem B), where the dimension of binary variables is 130 and the number of inequalities is 264.

We show the computational result. Let ${\underline{J}}^{*}$ denote the optimal value of the lower bound of a given cost function in Problem 1. Let ${\bar{J}}^{*}$ denote the upper bound of the cost function derived by using the optimal control input. First, consider the case of *ρ* = 10^−5^. Then, we can obtain ${\underline{J}}^{*}=2$ and ${\bar{J}}^{*}=15$, and *u* ^*s*^(*k*) is obtained as

(42) $$\begin{array}{c} u^{s}\left(0\right)=\cdots =u^{s}\left(4\right)=\left[\begin{array}{c} 1\\ 0 \end{array}\right]. \end{array}$$

Noting that *r*_2,1_ = 0.1 and *ρ* = 10^−5^( = 0.1^5^), all combinations of the Boolean functions are considered, and the value of *ρ* is not appropriate. In particular, ${\bar{J}}^{*}=15$ implies that *x*_1_(*k*) = 1, *k* = 0,1,…, 5, and is the trivial upper bound.

Next, consider the case of *ρ* = 0.2. Then, we can obtain ${\underline{J}}^{*}={\bar{J}}^{*}=4$, and *u* ^s^(*k*) is obtained as

(43) $$\begin{array}{c} u^{s}\left(0\right)=u^{s}\left(1\right)=\left[\begin{array}{c} 0\\ 1 \end{array}\right],\;u^{s}\left(2\right)=u^{s}\left(3\right)=u^{s}\left(4\right)=\left[\begin{array}{c} 1\\ 0 \end{array}\right]. \end{array}$$

From the obtained inputs, we see that the system is controlled by switching two discrete probability distributions, and the obtained inputs satisfy the constraint on duration of drug effectiveness. Noting that the trivial value of ${\bar{J}}^{*}$ is 15, we see that in this case the effectiveness of control synthesis is clear.

Finally, we discuss the computation time for solving Problem 1. The computation time of the ILP problem was less than 20 [msec], where we used IBM ILOG CPLEX 11.0 as an ILP solver on the computer with Windows Vista 32-bit, the Intel Core 2 Duo CPU 3.0 GHz, and the 4 GB memory. Since the WNT5A network considered here is small size, Problem 1 can be solved fast.

### 3.2. Artificial Example

In order to evaluate the computation time for solving Problem 1, we consider one artificial example of a BN with 15 states and 3 control inputs. We stress that the existing method [11, 12, 17–19, 21] cannot be applied to such a BN. This is because it is necessary to compute the state transition diagram such as that in Figure 2, that is, the transition probability matrix with 2^*n*^ × 2^*n*^. In naive implementation using MATLAB [34], matrices with 2^15^ × 2^15^ cannot be created due to memory consumption, where we used the computer described previously.

The optimal control problem is formulated. In this example, we consider 3 control inputs and 2 structural control inputs. If *u*_1_^*s*^(*k*) = 1, then the system is given as follows:

(44) $$\begin{array}{c} x\left(k+1\right)=\{\begin{array}{cc} f_{1}\left(x\left(k\right),u\left(k\right)\right) & \text{with}\;\text{the}\;\text{probability}\;0.8,\\ f_{2}\left(x\left(k\right),u\left(k\right)\right) & \text{with}\;\text{the}\;\text{probability}\;0.2. \end{array} \end{array}$$

If *u*_2_^*s*^(*k*) = 1, then the system is given as follows:

(45) $$\begin{array}{c} x\left(k+1\right)=\{\begin{array}{cc} f_{1}\left(x\left(k\right),u\left(k\right)\right) & \text{with}\;\text{the}\;\text{probability}\;0.2,\\ f_{2}\left(x\left(k\right),u\left(k\right)\right) & \text{with}\;\text{the}\;\text{probability}\;0.8. \end{array} \end{array}$$

The Boolean function *f*_1_ is given by the following:

(46) x1(k+1)=x1(k)∧¬x6(k)∨u3(k),x2(k+1)=¬x4(k)∧u1(k)∨u3(k),x3(k+1)=x5(k)∧u1(k)∨¬x10(k)∧x12(k)∧u3(k),x4(k+1)=x2(k)∧x5(k)∧¬u1(k)∨¬x14(k),x5(k+1)=¬u1(k)∧x6(k)∧x7(k)∨x12(k)∧x14(k),x6(k+1)=x1(k)∧x6(k)∧x10(k)∨¬x15(k),x7(k+1)=x6(k)∧x7(k)∧x8(k)∨u2(k)∧¬u3(k),x8(k+1)=x5(k)∧¬u1(k)∨x10(k)∧u2(k)∧x13(k),x9(k+1)=x3(k)∧u1(k)∨¬x8(k)∧x11(k),x10(k+1)=x6(k),x11(k+1)=x6(k)∧x10(k)∨¬u2(k)∧u3(k),x12(k+1)=x12(k)∧¬x15(k),x13(k+1)=¬u3(k),x14(k+1)=¬x14(k)∧u3(k),x15(k+1)=x14(k)∧x15(k).

The Boolean function *f*_2_ is given by the following:

(47) x1(k+1)=x2(k)∧x4(k)∧¬x8(k),x2(k+1)=¬x2(k)∧x3(k)∨u3(k),x3(k+1)=x1(k)∨¬x2(k)∧x3(k)∧x4(k),x4(k+1)=¬x1(k)∧x2(k)∧u1(k)∨x14(k),x5(k+1)=¬u2(k)∧x13(k)∧x14(k)∧x15(k),x6(k+1)=¬x2(k)∨x5(k)∧u1(k)∧¬u3(k),x7(k+1)=u1(k)∧x13(k),x8(k+1)=x5(k)∧x13(k),x9(k+1)=¬x6(k),x10(k+1)=x2(k)∧u2(k)∧¬x12(k)∧u3(k),x11(k+1)=¬x5(k)∧u1(k)∨¬x15(k)∧u3(k),x12(k+1)=x7(k)∧x3(k),x13(k+1)=x7(k)∧u2(k)∧u3(k),x14(k+1)=x12(k)∨x14(k)∧u3(k),x15(k+1)=x8(k).

From the previous setting, *r*_1,1_ = 0.8, *r*_1,2_ = 0.2, *r*_2,1_ = 0.2, *r*_2,2_ = 0.8 hold. In Problem 1, *Q*, *Q*_*f*_, *R*, and *R*_*s*_ are given as $Q=\left[\begin{array}{ccc} 1 & \cdots & 1 \end{array}\right]$, $Q_{f}=\left[\begin{array}{ccc} 10 & \cdots & 10 \end{array}\right]$, $R=\left[\begin{array}{ccc} 1 & 0 & 1 \end{array}\right]$, and $R_{s}=\left[\begin{array}{cc} 0 & 0 \end{array}\right]$, respectively. The initial state and the parameter *ρ* are given as $x_{0}={\left[\begin{array}{ccc} 1 & \cdots & 1 \end{array}\right]}^{T}$ and *ρ* = 10^−4^, respectively. The constraint on duration of drug effectiveness is imposed for *u*_1_(*k*) and *u*_2_^*s*^(*k*). For *u*_1_, the parameters *L*_*u*_1__, *W*_*u*_1__^1^, and *W*_*u*_1__^0^ are given as *L*_*u*_1__ = 0, *W*_*u*_1__^1^ = 3, and *W*_*u*_1__^0^ = 3, respectively. For *u*_2_^*s*^, the parameters *L*_*u*_2_^*s*^_, *W*_*u*_2_^*s*^_^1^, and *W*_*u*_2_^*s*^_^0^ are given as *L*_*u*_2_^*s*^_ = 1, *W*_*u*_2_^*s*^_^1^ = 3, and *W*_*u*_2_^*s*^_^0^ = 2, respectively.

Next, we discuss the computation time. Consider the two cases of *N* = 10 and *N* = 20. Then, in the ILP problem (Problem B) obtained, the dimension of binary variables is 1420 for *N* = 10 and 2840 for *N* = 20, and the number of inequalities is 3381 for *N* = 10 and 6731 for *N* = 20. In the case of *N* = 10, the computation time of the ILP problem was 96 [sec], where we used the computer described previously. In the case of *N* = 20, the computation time of the ILP problem was 238 [sec]. We remark that BNs with such a size are large scale in control problems of gene regulatory networks. Thus, we conclude that Problem 1 can be solved within the practical computation time.

## 4. Conclusions

In this paper, we have proposed a Boolean network (BN) model with two types of the control inputs and an optimal control method. By using this model, several situations in control of gene regulatory networks can be modeled. To model more realistic situations, duration of drug effectiveness has also been introduced. Since duration is given for each control input, effectiveness of multiple drugs can be evaluated. Furthermore, for this BN model, the optimal control problem has been formulated, and this problem is reduced to an integer linear programming problem. Finally, numerical simulations have been shown. The proposed method provides us with a basic in the control theory of gene regulatory networks.

Recently, to simplify state transition diagrams such as that in Figure 2, a stochastic Boolean network has been proposed in [35]. The authors proposed in [36] a similar method using polynomial optimization. In addition, to simplify a given Boolean network, the Karnaugh map realization of a Boolean network has been proposed in [37]. These methods are useful for reducing the computational burden. It is one of the future works to consider the control problem with duration of drug effectiveness based on these methods.
